# Analysis of abnormal posture in patients with Parkinson's disease using a computational model considering muscle tones

**DOI:** 10.3389/fncom.2023.1218707

**Published:** 2023-10-05

**Authors:** Yuichiro Omura, Hiroki Togo, Kohei Kaminishi, Tetsuya Hasegawa, Ryosuke Chiba, Arito Yozu, Kaoru Takakusaki, Mitsunari Abe, Yuji Takahashi, Takashi Hanakawa, Jun Ota

**Affiliations:** ^1^Department of Precision Engineering, School of Engineering, The University of Tokyo, Tokyo, Japan; ^2^Department of Integrated Neuroanatomy and Neuroimaging, Kyoto University Graduate School of Medicine and Faculty of Medicine, Kyoto, Japan; ^3^Department of Advanced Neuroimaging, Integrative Brain Imaging Center, National Center of Neurology and Psychiatry, Tokyo, Japan; ^4^Research into Artifacts, Center for Engineering, School of Engineering, The University of Tokyo, Tokyo, Japan; ^5^Division on Neuroscience, Department of Physiology, Asahikawa Medical University, Asahikawa, Japan; ^6^Department of Neurology, National Center Hospital, National Center of Neurology and Psychiatry, Tokyo, Japan

**Keywords:** Parkinson's disease, abnormal posture, computational model, musculoskeletal model, neural controller model

## Abstract

Patients with Parkinson's disease (PD) exhibit distinct abnormal postures, including neck-down, stooped postures, and Pisa syndrome, collectively termed “abnormal posture” henceforth. In the previous study, when assuming an upright stance, patients with PD exhibit heightened instability in contrast to healthy individuals with disturbance, implying that abnormal postures serve as compensatory mechanisms to mitigate sway during static standing. However, limited studies have explored the relationship between abnormal posture and sway in the context of static standing. Increased muscle tone (i.e., constant muscle activity against the gravity) has been proposed as an underlying reason for abnormal postures. Therefore, this study aimed to investigate the following hypothesis: abnormal posture with increased muscle tone leads to a smaller sway compared with that in other postures, including normal upright standing, under the sway minimization criterion. To investigate the hypothesis, we assessed the sway in multiple postures, which is determined by joint angles, including cases with bended hip joints. Our approach involved conducting forward dynamics simulations using a computational model comprising a musculoskeletal model and a neural controller model. The neural controller model proposed integrates two types of control mechanisms: feedforward control (representing muscle tone as a vector) and feedback control using proprioceptive and vestibular sensory information. An optimization was performed to determine the posture of the musculoskeletal model and the accompanied parameters of the neural controller model for each of the given muscle tone vector to minimize sway. The optimized postures to minimize sway for the optimal muscle tone vector of patients with PD were compared to the actual postures observed in these patients. The results revealed that on average, the joint-angle differences between these postures was <4°, which was less than one-tenth of the typical joint range of motion. These results suggest that patients with PD exhibit less sway in the abnormal posture than in other postures. Thus, adopting an abnormal posture with increased muscle tone can potentially serve as a valid strategy for minimizing sway in patients with PD.

## 1. Introduction

Parkinson's disease (PD) is a neurodegenerative disorder that primarily affects patients aged >60 years. The incidence of PD increases alongside the aging population (Van Den Eeden et al., 2003; Poewe et al., 2017). The motor symptoms of PD encompass rigidity and postural instability (Balestrino and Schapira, 2020), with the former being linked to increased muscle tone, which is continuous muscle activity against gravitational forces (Doherty et al., 2011; Ivanenko and Gurfinkel, 2018).

Patients with PD exhibit a distinct abnormal posture during standing, typified by conditions such as neck-down and stooped postures and Pisa syndrome (Doherty et al., 2011). These postures often cause back pain and compromise lung capacity, exerting a substantial influence on overall quality of life of patients (Bloch et al., 2006; Doherty et al., 2011). Moreover, these abnormal postures vary among patients with PD, presenting a range of postural configurations collectively categorized as abnormal posture. The pathology underlying these abnormal postures remains ambiguous, prompting the formulation of multiple hypotheses. One prevailing hypothesis points toward rigidity (Doherty et al., 2011). Studies investigating the interplay between the truncal muscles and motor symptoms have reported that hypertonia within the truncal muscles could potentially contribute to postural deficits (Wright et al., 2007). Additionally, studies have shown that lidocaine administration to the external oblique muscle ameliorates abnormal postures, thereby implying that dystonia within the truncal flexor muscles plays a role in the emergence of these abnormal postures (Furusawa et al., 2015). These results underscore a conceivable connection between abnormal posture and increased muscle tone.

Notably, a relationship between abnormal postures and postural sway during standing has also been reported. Bloem et al. proposed that an abnormal posture serves as a compensatory mechanism to avert backward falls by stabilizing the standing posture against foot rotation, such as toe lifting (Bloem et al., 1999). Dietz et al. examined the sway of patients with PD standing on a treadmill who were subjected to anteroposterior sinusoidal movement and reported that the patients exhibited increased sway in upright postures compared with the control subjects, and the upper body of the former failed to adequately adapt to the imposed vibration (Dietz et al., 1993). Conversely, Jacobs et al. observed a reduction in the stability margin [i.e., the difference between the center of mass (CoM) and center of pressure (CoP)] when healthy individuals assumed an abnormal posture, suggesting that individuals who adopt an abnormal posture are more prone to falls when exposed to disturbances (Jacobs et al., 2005). This is inconsistent with the idea that abnormal posture compensates for fall prevention (Jacobs et al., 2005). However, in light of the aforementioned experiments conducted by Dietz et al., it was determined that under perturbed conditions, patients with PD in an upright posture exhibit greater sway than healthy individuals standing in an upright posture (Dietz et al., 1993). Thus, patients with PD may be able to stand with less sway in an abnormal posture than in an upright posture.

Minimizing sway is one of the objectives of human postural control during static standing. To achieve this, one approach involves adjusting the positions of the CoP and CoM to remain within the base of support (Massion et al., 2004; Horak, 2006). However, even when the position of the CoM is within the base of support, a high CoM velocity can lead to instability and potential falling. Therefore, CoM velocity is equally important to keeping the CoM within the base of support to maintain balance while standing (Pai and Patton, 1997). Based on these findings, in the case of patients with PD exhibiting increased muscle tone, sway in abnormal postures (particularly CoM velocity) may be less pronounced than in other postures. However, studies regarding abnormal postures and sway during static standing are limited, and the aforementioned studies only focused on abnormal posture and sway under disturbance.

Given that patients with PD often display abnormal postures even in the absence of external perturbation while static standing, it is important to uncover the relationship between such abnormal postures and postural sway during static standing to understand abnormal postures in depth and apply corrective measures. Hence, this study aimed to scrutinize the hypotheses concerning the association between abnormal postures and postural sway during static standing. As previously noted, existing research has hinted at a connection between abnormal posture, increased muscle tone, and postural sway (Dietz et al., 1993; Bloem et al., 1999; Jacobs et al., 2005; Doherty et al., 2011). Therefore, this study examined the following hypothesis: abnormal posture with increased muscle tone leads to smaller sway compared with that in other postures, including normal upright standing, under the sway minimization criterion.

## 2. Methods

### 2.1. Overview

To assess the abovementioned hypothesis, it is essential to evaluate the extent of postural sway across diverse standing postures determined via the combinations of joint angles in the presence of increased muscle tone. Herein, we performed forward dynamics simulations involving a computational model encompassing a musculoskeletal model representing the human body and a neural controller model responsible for regulating the musculoskeletal model while also representing muscle tone. This model enables the determination of postural sway magnitude for various given sets of parameters, including overall body muscle tone, posture of the musculoskeletal model (joint angle), and parameters of the neural controller model.

While muscle tone might be elevated in patients with PD, the precise measurement of comprehensive whole-body muscle tone in such patients is challenging. To tackle this concern, the concept of muscle tone vector is introduced, which represents the muscle tone across the entire body, to accomplish the study objective through the following approaches:

1. We calculated the muscle tone vector for each patient with PD so that the simulated posture and sway could reproduce the experimental posture and sway.
2. We compared the simulated postures at the calculated muscle tone vectors with the experimental postures.

### 2.2. Problem setting

#### 2.2.1. Tasks

Herein, we focused on the abnormal postures and postural sways observed in patients with PD during static standing. Consequently, the objective assigned to the computational model is to maintain a static standing posture for 5 s. Scaling and forward dynamics simulations were performed on the musculoskeletal simulators, OpenSim (Delp et al., 2007; Seth et al., 2018) and SCONE (Hyfydy) (Geijtenbeek, 2019, 2021).

#### 2.2.2. Attempts

We made the following assumptions about human postural control.

- One of the objectives of human postural control is minimizing of the CoM velocity during static standing.

The aims of human postural control encompass the manipulation of both the positions of the CoM and CoP to ensure that they are within the base of support (Massion et al., 2004; Horak, 2006). Studies have highlighted the significance of the position and velocity of CoM as an escalation in the velocity poses a challenge to maintaining equilibrium (Pai and Patton, 1997). Furthermore, it has been proposed that among the CoM position, velocity, and acceleration utilized for stabilizing human postures during static standing, CoM velocity is the most precise indicator (Jeka et al., 2004). This perspective underscores the vital role of CoM velocity within postural control in static standing. Accordingly, the objectives of human postural control include maintaining the CoM position within the base of support and minimizing the CoM velocity.

### 2.3. Computational model

**Figure 1:**
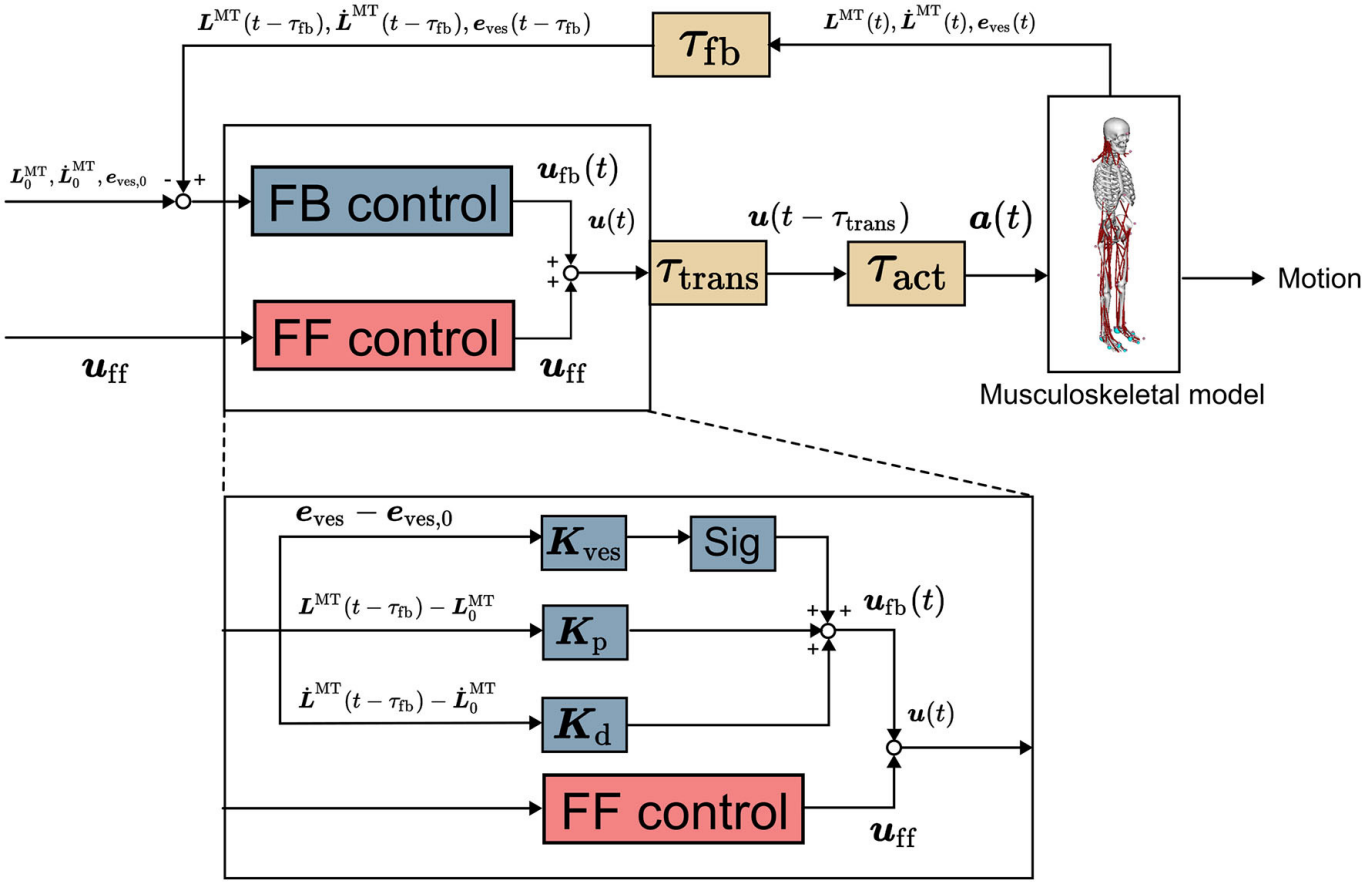
Computational model (Omura et al., 2022). It comprises musculoskeletal and neural controller models involving feedforward control, feedback control, and time delay. ***u***_ff_(*t*), ***u***_fb_(*t*), and ***u***(*t*) are vectors representing the signals of the FF and FB controls and their sum for all the muscles at *t* seconds, respectively. ***K***_p_, ***K***_d_, and ***K***_ves_ are the FB gains. ***L***^MT^(*t*) and ${\dot{L}}^{\text{MT}}\left(t\right)$ are the muscle length and stretch rate at *t* seconds, respectively, with subscript 0 representing the initial value. ***e***_ves_ is a vector representing vestibular sensory information. *sig* is 1 for an extensor muscle and −1 for a flexor muscle, providing an excitatory signal to the extensor and an inhibitory signal to the flexor. ***a***(*t*) is the muscle activity at *t* seconds.

This section delineates the computational model utilized in the present study. An overview of the model is depicted in Figure 1. Further details concerning the computational model can be found in our previous publication (Omura et al., 2022).

#### 2.3.1. Musculoskeletal model

We utilized a musculoskeletal model encompassing 21 degrees of freedom (DOF) and 94 muscles, as proposed in our study, to represent the human body (Omura et al., 2022). Typical abnormal postures among patients with PD encompass hip and knee flexion, while severely abnormal posture also includes neck flexion (Doherty et al., 2011). In conjunction with these DOFs, the musculoskeletal model incorporates joint DOF for the back and ankles. The musculoskeletal model with these joint DOF can reproduce the abnormal postures of patients with PD. The musculoskeletal model was scaled to the motion capture data described below.

#### 2.3.2. Neural controller model

The neural controller model proposed in our study was used to control the musculoskeletal model (Omura et al., 2022). In the previous study, this neural controller model was utilized to replicate human postural sway, and the reproduced sway was valid as that of humans (Omura et al., 2022). The neural controller model comprises an FF control mechanism to model muscle tones and an FB control mechanism employing proprioceptive and vestibular information and accounts for time delays as well. These control mechanisms were implemented based on the reticulospinal and vestibulospinal tracts, which are important for human postural control (Takakusaki, 2017; Omura et al., 2022). The FB controller operates with a time delay owing to multiple sources of delay, including synaptic and muscle-activation dynamics as well as signal transmission along sensory fibers (τ_*fb*_). The summation of time delay is up to 120 ms (Zajac, 1989; Winters, 1995; Masani et al., 2006). The output of the neural controller model directed to the *i*th muscle is expressed as follows (Jiang et al., 2016; Omura et al., 2022).


(1) $$\begin{array}{l} u_{i}=u_{\text{ff},\text{i}}+u_{\text{fb},\text{i}} \end{array}$$

(2) $$\begin{array}{l} u_{\text{fb},\text{i}}=u_{\text{fb},\text{prop},\text{i}}+u_{\text{fb},\text{ves},\text{i}} \end{array}$$

(3) $$\begin{array}{l} u_{\text{fb},\text{prop}}=K_{\text{p},\text{i}}\frac{L_{i}^{\text{MT}}\left(t-\tau_{\text{fb}}\right)-L_{\text{i},0}^{\text{MT}}}{L_{\text{i},0}^{\text{MT}}}+K_{\text{d},\text{i}}\frac{{\dot{L}}_{i}^{\text{MT}}\left(t-\tau_{\text{fb}}\right)-{\dot{L}}_{\text{i},0}^{\text{MT}}}{V_{\text{max}}} \end{array}$$

(4) $$\begin{array}{l} u_{\text{fb},\text{ves},\text{i}}=sigK_{\text{ves},\text{i}}e_{\text{ves}} \end{array}$$

*u*_ff,i_ and *u*_fb,i_ are the FF and FB outputs to the *i*th muscle, respectively. *u*_fb,prop,i_ and *u*_fb,ves,i_ are the FB outputs using proprioceptive and vestibular information to the *i*th muscle, respectively. *K*_p,i_ and *K*_d,i_ are the FB gains of the *i*th muscle, respectively. ***K***_ves,i_ is the vector of the FB gain vector of the *i*th muscle, respectively. $L_{i}^{\text{MT}}$ and ${\dot{L}}_{i}^{\text{MT}}$ are the length and lengthening speed of the *i*th muscle. $L_{\text{i},0}^{\text{MT}}$ and ${\dot{L}}_{\text{i},0}^{\text{MT}}$ are the initial muscle length and lengthening speed of the *i*th muscle. τ_fb_ is the time delay due to the signal transmission along sensory fibers (τ_fb_ = 40*ms*). The *sig* outputs an excitatory signal to the extensors and an inhibitory signal to the flexors by being 1 for extensors and −1 for flexors. ***e***_ves_ is the vector that represents the deviations of vestibular information from the target values.

Let ***u***_ff_, a vector with *u*_ff,i_ in Equation (1) as each element, be a muscle tone vector. The muscle tone vector can have various values even for the same posture. $\|u_{\text{ff}}|{|}^{2}$, the square of the norm of the muscle tone vector, represents the index of the height of the muscle tone of the whole body, which was obtained from the previous study for comparison (Jiang et al., 2016). A large value of this index indicates a high muscle tone throughout the body. *u*_fb,prop,i_ is the output of the FB control using the muscle length and lengthening speed of the musculoskeletal model as the FB information to the *i*th muscle. *u*_fb,prop,i_ is the difference from the initial value of each FB information ($L_{\text{i},0}^{\text{MT}}$ and ${\dot{L}}_{\text{i},0}^{\text{MT}}$, which are the initial muscle length and lengthening speed of the *i*th muscle, respectively) multiplied by the FB gains, as shown in Equation (3). *u*_fb,ves,i_ is the output of the FB control using the position information (position, angle, velocity, angular velocity, acceleration, and angular acceleration) of the head of the musculoskeletal model as the FB information. Moreover, *u*_fb,ves,i_ is the difference from the target value of each FB information multiplied by the FB gains, as expressed in Equation (3). See our previous papers for details (Omura et al., 2022).

### 2.4. Posture calculation

**Figure 2:**
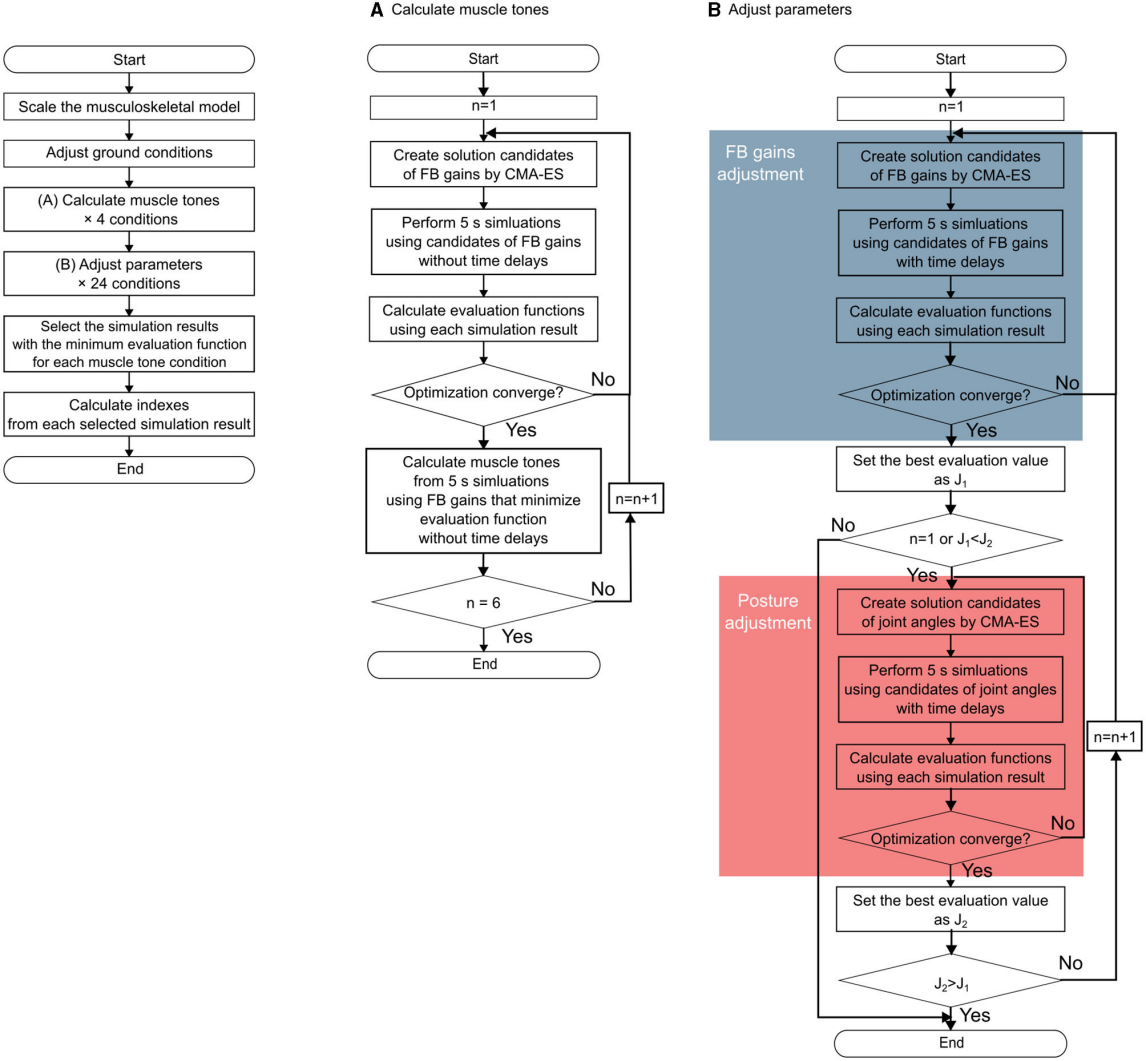
Flowchart of the entire method. The left part of the figure shows the outline of the entire method of the data of one participant. **(A)** Outline of the muscle tone vector estimation. **(B)** Outline of the parameter adjustment procedure. The blue part shows the procedure for the feedback gain adjustment, and the red part shows the procedure for the posture (target posture) adjustment.

This section outlines the approach employed to identify postures using a computational model. Specifically, our approach encompasses the following steps: (i) calculating candidates for muscle tone vectors with varying norms corresponding to the postures of patients with PD and (ii) for each candidate of muscle tone vector, determining the posture of the musculoskeletal model and parameters of the neural controller model. These parameters are calculated to ensure a static standing with minimal sway, which was accomplished through an optimization method. The methodological overview is visually depicted in Figure 2.

The optimization method employed was the covariance matrix adaptation evolution strategy (CMA-ES), according to the previous study (Jiang et al., 2016). This method can be used to find postures with less noticeable swaying at different muscle tone vectors.

#### 2.4.1. Measurement of the posture of patients with PD

We utilized marker data derived from the standing posture of patients with PD measured via a motion capture system.

The analysis included eight patients with PD (4 men and 4 women, aged 66.9 ± 8.4 years). Participant information is present in Table 1. We attached 31 or 59 markers to the entire body of the participants. The measurements were conducted during static standing for 5 s using a motion capture system (6-camera, Oqus 3+/Oqus 5+, Qualisys, Gothenburg, Sweden). One trial involved the patient standing static for 5 s. Each participant performed this trial twice or thrice. The studies involving human participants were reviewed and approved by the Ethics Committee at the National Center of Neurology and Psychiatry (A2019-126). The patients provided their written informed consent to participate in this study. We analyzed the first trial with no measurement defects among the two or three trials. Additionally, in the analysis of static standing, the measurement data of the initial 4 s of the entire 5 s were considered owing to some deficiencies in the last 1 s, such as movement exhibited by several participants. In the case of Sub3, the marker position measurements were incomplete during the first 0.25 s, thereby necessitating the exclusion of this segment from our analysis.

**Table 1:** Data obtained from patients with PD.

**Participants**	**Age**	**H&Y scale**	**UPDRS part III**
Sub1	72	3	26
Sub2	79	3	21
Sub3	66	3	24
Sub4	65	2	48
Sub5	73	2	19
Sub6	69	2	26
Sub7	53	2	17
Sub8	58	2	28

To scale the musculoskeletal model, a set of 23 or 27 marker data points was employed. These markers encompassed the anterior and posterior superior iliac spines, greater trochanter, medial and lateral femoral epicondyles, medial and lateral malleoli, calcanei, 1st and 5th metatarsal heads, acromion processes, head, C7 vertebrae, TH12, episternum, and xiphoid process of the sternum. To scale the musculoskeletal model for each patient, the postures of the patients were replicated using a 2 s duration of marker data. This choice of a 2 s for scaling was motivated by the potential for error escalation if the posture lacked stability and encompassed swaying. For scaling and reproduction, we used the scaling and inverse kinematics (IK) tools of OpenSim (Delp et al., 2007; Seth et al., 2018). Scaling was executed by adapting the distances between markers on the musculoskeletal model according to the corresponding markers on patients with PD. The subsequent computation of body weight relied on the ideal body weight calculations (BMI: 22) (Tokunaga et al., 1991) based on the height derived from the scaling outcome. Employing IK alongside marker data, the joint angles of the musculoskeletal model were computed. This ensured congruence between every marker on the model and their corresponding counterparts in patients with PD. By adopting this methodology, the sway exhibited by each patient with PD was reproduced on the musculoskeletal model.

The posture replicated through IK involved issues related to ground contact due to disparities between the marker positions within the musculoskeletal model and those observed in patients with PD. This discrepancy led to instances where the model's feet appeared to hover or were embedded within the floor. These challenges had adverse implications; for instance, they precipitated occurrences where the musculoskeletal model either collapsed or instantly emerged at the onset of forward dynamics simulations. Consequently, the joint angles of the lower limbs were adjusted via an optimization method to rectify the ground contact issue between the feet and the floor of the posture reproduced by IK. The following evaluation function sought to minimize alterations in the CoM position of the feet to obtain an appropriate posture. The optimization method employed for this purpose was the CMA-ES, akin to its application in the adjustment of the posture and FB gains within the neural controller model, which has been elaborated upon subsequently. CMA-ES is an effective evolution strategy for non-linear and non-convex functions that do not require derivatives (Hansen, 2016). Therefore, CMA-ES was employed to adjust the posture and FB gains, which are non-linear and have derivatives that are difficult to calculate.


(5) $$\begin{array}{l} J_{\text{pos}}=w_{\text{force}}J_{\text{force}}+w_{\text{COMpos}}J_{\text{COMpos}}+w_{\text{feetvel}}J_{\text{feetvel}} \end{array}$$

(6) $$\begin{array}{l} J_{\text{force}}=\int_{0}^{0.1}\left(\left|f_{\text{r}}\left(t\right)-f_{\text{r}}\left(0\right)|+|f_{\text{l}}\left(t\right)-f_{\text{l}}\left(0\right)\right|\right)dt \end{array}$$

(7) $$\begin{array}{l} J_{\text{COMpos}}=|x_{\text{COM},\text{l}}-x_{\text{COM},\text{exp},\text{l}}|+|x_{\text{COM},\text{r}}-x_{\text{COM},\text{exp},\text{r}}| \end{array}$$

(8) $$\begin{array}{l} J_{\text{feetvel}}=(\int_{0}^{0.1}|v_{\text{ankle},\text{r}}(t)|+|v_{\text{ankle},\text{l}}(t)|+|v_{\text{subtalar},\text{r}}(t)|\\ \;+\left|v_{\text{subtalar},\text{l}}(t)\right|)dt \end{array}$$

*J*_force_ is a term that evaluates the magnitude of the floor reaction force. *f*(*t*) denotes the floor reaction force in *t* seconds. The subscripts r and l denote the right and left foot, respectively. *J*_COMpos_ is a term that evaluates the CoM position relative to the foot. *x*_COM_ and *x*_COM,exp_ denote the CoM position relative to the foot at 0 s and the CoM position relative to the foot as reproduced by IK, respectively. *J*_feetvel_ is a term that evaluates the angular velocity of the joints around the foot. *v*_ankle_(*t*) and *v*_subtalar_(*t*) denote the angular velocities of the ankle and subtalar joints in *t* seconds, respectively.

#### 2.4.2. Calculation of candidates for muscle tone vector

To determine the candidates of the muscle tone vector associated with the measured posture of each patient with PD, we first aligned the posture of the musculoskeletal model to replicate that of the patient. The candidates for the muscle tone vector were derived using a musculoskeletal model, scaled to the posture of a patient with PD. The procedural framework is depicted in Figure 2A. Muscle tone indicates constant muscle activity and is independent of time delay. Therefore, the experimental posture was set to the posture of the musculoskeletal model and the posture was maintained for 5 s under the condition without time delay and with FB control only, based on previous studies (Jiang et al., 2016). From these results, the average muscle activity of each muscle in the second half was calculated as the muscle tone. The muscle tone was obtained using the following equation (Jiang et al., 2016):


(9) $$\begin{array}{l} u_{\text{ff},\text{i}}=\frac{\int_{t_{1}}^{t_{2}}a_{i}\left(t\right)dt}{t_{2}-t_{1}} \end{array}$$

*u*_ff,i_ denotes the muscle tone in the *i*th muscle. *a*_*i*_(*t*) denotes the muscle activity of the *i*th muscle in *t* seconds. *t*_1_ and *t*_2_ are 3 and 5 s, respectively, based on the previous study (Jiang et al., 2016). The muscle tone vector is given in the following equation:


(10) $$\begin{array}{l} u_{\text{ff}}=\left[\begin{array}{cccc} u_{\text{ff},1} & u_{\text{ff},2} & \cdots & u_{\text{ff},94} \end{array}\right] \end{array}$$

The muscle tone vectors that could maintain the standing posture for 5 s for each given posture were not unique and different muscle tone vectors could do the same job depending on the values of FB gains used for the simulations. Furthermore, the muscle tone vectors procured might not necessarily ensure the model's ability to maintain a standing posture under circumstances involving time delays. Moreover, the task of measuring muscle tone throughout the entire body of actual patients with PD presents inherent challenges. Therefore, multiple muscle tone vectors need to be calculated as candidates, from which we can narrow down a list of muscle tone vectors to the “optimal” one.

To calculate candidates for muscle tone vector, the given FB gains were determined via the CMA-ES. It is possible to calculate muscle tone vectors that have different values by incorporating the cosine similarity with the already calculated muscle tone vectors into the evaluation function. To calculate the muscle tone vector with arbitrary $\|u_{\text{ff}}|{|}^{2}$, a term evaluating $\|u_{\text{ff}}|{|}^{2}$ is added to the evaluation function. In addition, two terms for evaluating falls and the CoM velocity based on assumptions were incorporated. Thus, the evaluation function is expressed as follows:


(11) $$\begin{array}{l} J_{\text{mt}}=w_{\text{fall}}J_{\text{fall}}+w_{\text{com}}J_{\text{com}}+w_{\text{cos}}J_{\text{cos}}+w_{{\text{u}}_{\text{ff}}}J_{{\text{u}}_{\text{ff}}} \end{array}$$

(12) $$\begin{array}{l} J_{\text{fall}}=\frac{1}{T_{\text{fail}}}\left(T_{\text{simu}}-T_{\text{fail}}\right) \end{array}$$

(13) $$\begin{array}{l} J_{\text{com}}=\frac{\int \left(v_{\text{com},\text{ x}}{\left(t\right)}^{2}+v_{\text{com},\text{ y}}{\left(t\right)}^{2}+v_{\text{com},\text{ z}}{\left(t\right)}^{2}\right)dt}{T_{\text{fall}}} \end{array}$$

(14) $$\begin{array}{l} J_{\text{cos}}=\frac{1}{n}\overset{n}{\underset{i=1}{\sum }}\frac{a_{i}\cdot b}{\left\|a_{i}\|\|b\right\|} \end{array}$$

(15) $$\begin{array}{l} J_{u_{\text{ff}}}=\|b|{|}^{2}-\alpha \end{array}$$

(16) $$\begin{array}{l} {w{\;}_{\text{u}}}_{\text{ff}}=\{\begin{array}{c} 1\;\left(|J_{{\text{u}}_{\text{ff}}}|<0.5\right)\\ 1000\;\left(|J_{{\text{u}}_{\text{ff}}}|\geq 0.5\right) \end{array} \end{array}$$

*J*_fall_ is a term that evaluates the time to fall. *T*_fail_ and *T*_simu_ denote the time to fall and the simulation time (5 s), respectively. *J*_com_ is a term that evaluates the CoM velocity. *v*_com,x_(*t*), *v*_com,y_(*t*), and *v*_com,z_(*t*) denote the CoM velocities in the x, y, and z axes in *t* seconds, respectively. *J*_cos_ is a term that evaluates the cosine similarity. *n* denotes the number of muscle tone vectors calculated so far. ***a***_*i*_ denotes the *i*th calculated muscle tone vector. *b* denotes the muscle tone vector to be calculated. *J*__**u**_ff_ is a term that evaluates $\|u_{\text{ff}}|{|}^{2}$ of the muscle tone vector. α denotes $\|u_{\text{ff}}|{|}^{2}$ of the muscle tone vector to be calculated. *w*_fall_, *w*_com_, *w*_uff_ and *w*_cos_ are 50,000,000, 5,000, 1,000, and 0.1, respectively, based on previous research, to search for solutions that do not fall and reduce the CoM speed preferentially (Kaminishi et al., 2019). *w*_uff_ was considered according to Equation (16), and the value of the term to evaluate the cosine similarity relatively increased when the muscle tone vector was calculated to ensure that the difference between the specified value and $\|u_{\text{ff}}|{|}^{2}$ was < 0.5. Consequently, muscle tone vectors with varying cosine similarities can be preferentially searched when the condition of $\|u_{\text{ff}}|{|}^{2}$ is fulfilled.

In a previous study employing a comparable computational model, muscle tone vectors were computed to achieve equidistant values of $\|u_{\text{ff}}|{|}^{2}$. The resulting muscle tone vectors were then compared with the experimental results of healthy individuals. This analysis unveiled that the muscle tone vector corresponding to $\|u_{\text{ff}}|{|}^{2}=2.07$ yielded muscle activity most akin to that of healthy individuals in the upright stance (Jiang et al., 2016). Herein, muscle tone vectors were calculated for $\|u_{\text{ff}}|{|}^{2}$ values exceeding $\|u_{\text{ff}}|{|}^{2}=2.07$. Through the adjustment of α in Equation 15, specifically setting it at 2, 4, 6, and 8, the computation encompassed the derivation of muscle tone vectors with $\|u_{\text{ff}}|{|}^{2}$ values lying within a range of ±0.5 from these designated magnitudes. To calculate muscle tone vectors with multiple directions, we repeatedly computed the muscle tone vector until *n* = 6 at Equation (14) empirically (Figure 2A).

#### 2.4.3. FB gains and posture calculation

Using the candidates of muscle tone vectors in the postures of patients with PD that were calculated in the previous section, we searched for a posture in which the musculoskeletal model can maintain a standing posture with minimal sway under conditions with a time delay. To achieve this, the parameters of the computational model were adjusted using the optimization method. The procedure is shown in Figure 2B. The target parameters are the FB gains and posture (target posture for FB control). We utilized the posture data of a patient with Parkinson's Disease as the starting point. From there, we used forward dynamics simulation to recreate sway and evaluated the recreated sway by adjusting the parameters accordingly. The FB gains were adjusted to maintain the standing posture despite time delays, and the posture of the musculoskeletal model was modified to find a posture with less sway. CMA-ES was used as the optimization method. The evaluation function is expressed as the following function that evaluates the CoM velocity based on the assumption.


(17) $$\begin{array}{l} J_{\text{param}}=w_{\text{fall}}J_{\text{fall}}+w_{\text{com}}J_{\text{com}} \end{array}$$

The FB gains encompass a total of 280 parameters, while posture involves 21 parameters in total. Simultaneously adjusting these parameters would lead to an expansive solution space owing to their high count.

To navigate this challenge, these parameters are alternately adjusted by tuning the FB gain and posture. The optimization method seeks to find the optimal values for these parameters. In the FB gain optimization, the optimal value of the evaluation function is denoted as *J*_1_, whereas in the optimization of the posture, it is denoted as *J*_2_. Each optimization iteration continues until *J*_1_ becomes < *J*_2_ during FB gain optimization or until *J*_2_ becomes < *J*_1_ during posture optimization.

We adjusted the parameters for each muscle tone vector that was calculated. To evaluate the posture and sway, we used the muscle tone vector with the smallest evaluation in each $\|u_{\text{ff}}|{|}^{2}$. This resulted in obtaining simulation results with optimized parameters for 32 conditions, which included 8 participants and 4 muscle tone vectors each.

### 2.5. Evaluation

The resulting posture and sway from the simulation using the optimized parameters were evaluated using the following procedure. The procedures correspond to the approach (1) and (2) in Section 2.1..

1. We selected the muscle tone vector for each patient with PD so that the simulated posture and sway could reproduce the experimental posture and sway.
2. We compared the simulated postures at the calculated muscle tone vectors with the experimental postures.

To assess and compare the standing posture and sway as described in (1), several metrics were calculated: (a) mean difference across all joint angles, (b) difference in the mean CoM velocity, and (c) standard deviation of all the joint angles. The evaluation of differences in the standing posture was quantitatively performed via (a), while (b) and (c) were employed to evaluate sway. The difference in the mean CoM velocity indicated by metric (b) was used to assess sway, grounded in certain assumptions. However, (b) might be inadequate to gauge the sway of individual body parts such as in cases where two body parts sway in opposing phases. Consequently, (c) was introduced to evaluate the sway of each specific body part.

As these metrics possess distinct units, each value was standardized using the T-score. The aggregate of squared T-scores was employed as an indicator of posture and sway. The muscle tone vector that yields the smallest index value for the combined posture and sway evaluation can be deemed the most optimal. Herein, we considered it as the muscle tone vector for the patients with PD, established through the optimization method. During step (2), we used (a) to compare the standing posture at the selected muscle tone vector with the experimental posture.

## 3. Results

### 3.1. Posture adjustment

**Figure 3:**
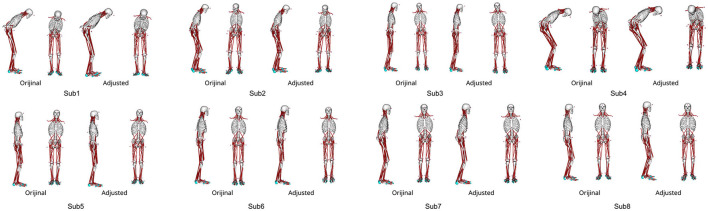
The abnormal posture of each participant before and after postural adjustment. Different postures are represented for each participant.

Figure 3 shows the patient postures reproduced from the measured marker data using IK along with the outcomes of the posture adjustment process described in 2.5. Notably, Subs 1 and 4 exhibited pronounced trunk flexion. For detailed variations in joint angles before and after posture adjustment, please refer to the Appendix in Supplementary material.

### 3.2. Parameter adjustment

For each participant, six potential candidates were computed for muscle tone vectors corresponding to α = 2, 4, 6, and 8. In other words, we could calculate the candidates for muscle tone vectors with distinct elements using cosine similarities.

For every muscle tone vector, the parameters were adjusted according to Section 2.4.3. Consequently, across all $\|u_{\text{ff}}|{|}^{2}$ values for each participant, the musculoskeletal model was able to maintain the standing posture for a minimum of 5 s employing at least one muscle tone vector. This suggests that the posture and FB gain necessary for maintaining a standing posture with time delays can be determined through parameter adjustment using the optimization method.

### 3.3. Muscle tones

**Figure 4:**
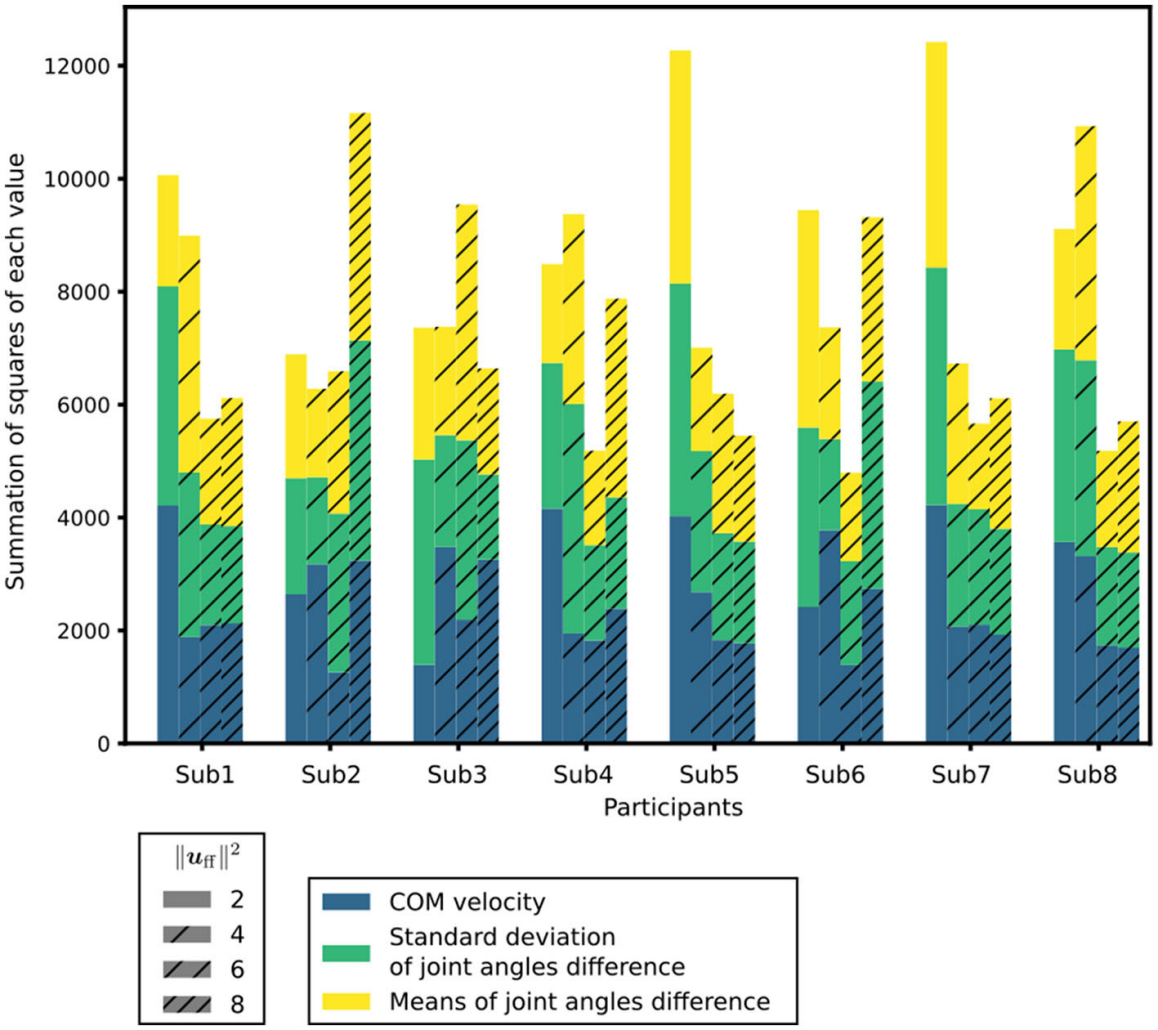
Index to evaluate the posture and sway under each condition. $\|u_{\text{ff}}|{|}^{2}$ is a measure of the muscle tone vector. In each condition, the differences in the mean CoM velocities, standard deviations of the joint angles, and mean T-score of the differences in the joint angles are shown in different colors.

The index for assessing posture and sway, as detailed in Section 2.5, was computed for each $\|u_{\text{ff}}|{|}^{2}$ condition. The indices to evaluate posture and sway under each condition are depicted in Figure 4. Among the participants, the muscle tone vectors yielding the smallest index were $\|u_{\text{ff}}|{|}^{2}=4$ for Sub2, $\|u_{\text{ff}}|{|}^{2}=6$ for Subs1, 4, 6, 7, and 8, and $\|u_{\text{ff}}|{|}^{2}=8$ for Subs3 and 5. These results indicate that the postural sway in these muscle tone vectors closely matches the experimental results. In other words, the posture and sway observed in these muscle tone vectors were the most representative of actual patients with PD. Previous studies utilizing computational models (Jiang et al., 2016) have suggested that a muscle tone vector with $\|u_{\text{ff}}|{|}^{2}$ = 2.07 is most fitting for healthy subjects. Notably, $\|u_{\text{ff}}|{|}^{2}$ = 4, 6, 8 exceed $\|u_{\text{ff}}|{|}^{2}$ = 2.07. Consequently, within the set of calculated muscle tone vectors ($\|u_{\text{ff}}|{|}^{2}$ = 2, 4, 6, 8), the muscle tone vector with a larger $\|u_{\text{ff}}|{|}^{2}$ than that of healthy individuals yielded the most suitable posture and sway for patients with PD. These outcomes are consistent with the observation that muscle tone is elevated in patients with PD compared with that in healthy individuals (Burleigh et al., 1995).

### 3.4. Comparing postures

**Figure 5:**
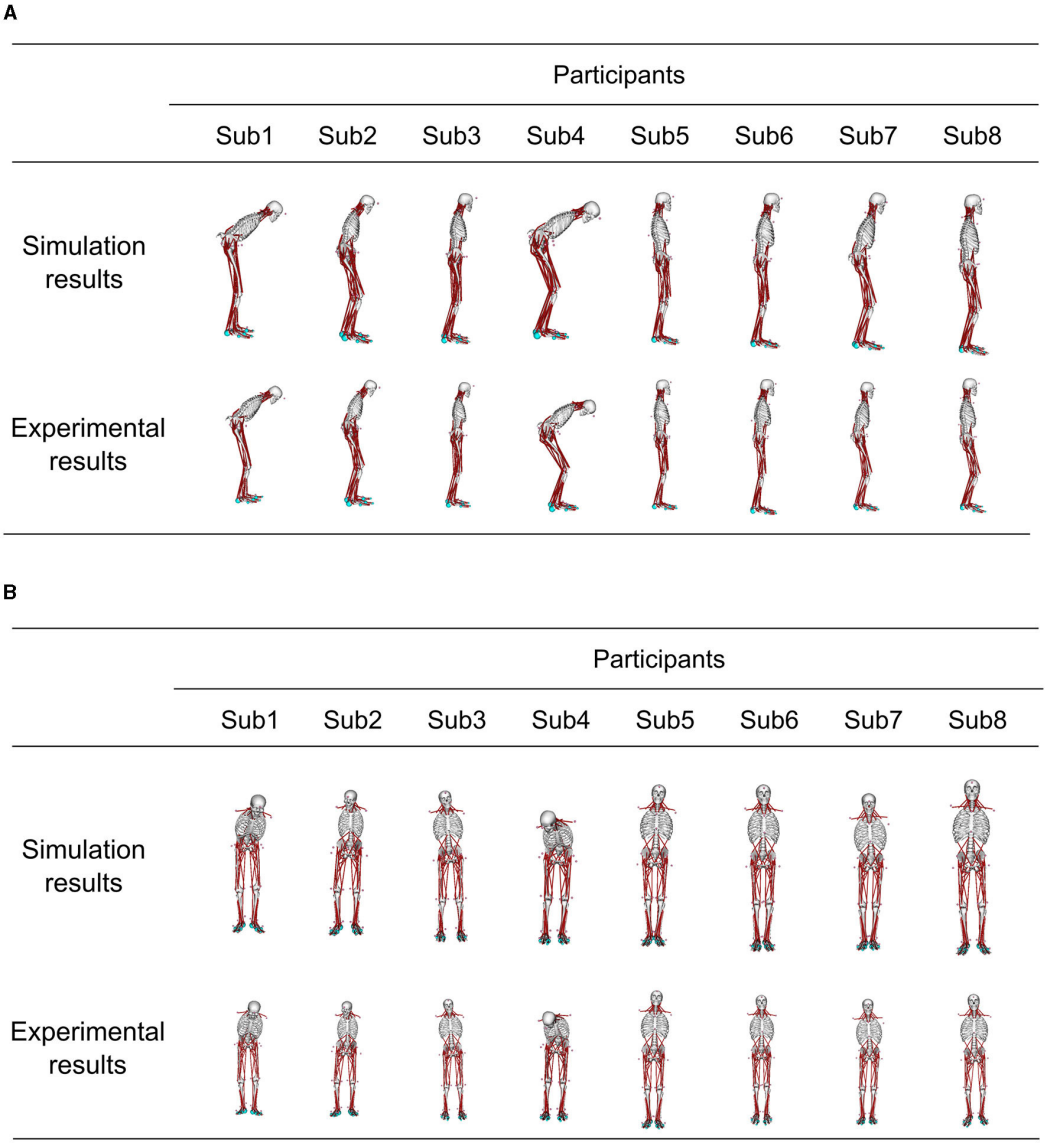
Comparison of the standing posture in the simulation results obtained using optimized parameters and the posture in the experiment results at the muscle tone vector where the index to evaluate the posture and sway in each participant was the smallest. The simulation result shows the average posture in 1–4 s. **(A)** Sagittal plane posture, and **(B)** frontal plane posture.

Figure 5 demonstrates the posture at the muscle tone vector corresponding to the minimum index for evaluating posture and sway in Figure 4, juxtaposed with the posture from experimental results. This depiction presents postures within the sagittal and frontal planes. The simulation result with optimized parameters represents the average standing posture from 1 to 4 s.

Furthermore, Table 2 provides mean values ± standard deviation (SD) of the joint angle differences for these postures. The comparison of these postures revealed that the mean joint-angle difference was <4° in all the participants except Sub4 where it was 5.2°. Calculating the mean + 2SD corresponding to a 95% confidence interval under the assumption of a normal distribution of joint angle differences revealed a mean + 2SD of 5.5° for Sub1 and 8.8° for Sub4.

**Table 2:** The mean value of all the joint-angle differences between the simulation results with optimized parameters and the experimental results at the muscle tone vector where the index to evaluate the posture and sway was the smallest ± standard deviations.

**Participants**	** $\|u_{\text{ff}}{\|}^{2}$ **	**Means of joints difference [deg]**
Sub1	6	2.7 ± 1.4
Sub2	4	1.4 ± 0.2
Sub3	8	2.5 ± 0.4
Sub4	8	5.2 ± 1.8
Sub5	8	2.6 ± 0.4
Sub6	6	0.9 ± 0.4
Sub7	8	2.2 ± 1.1
Sub8	6	1.8 ± 0.3

$\|u_{\text{ff}}|{|}^{2}$ represents the index of the muscle tone vector.

To summarize, a quantitative assessment of posture differences using joint angles (Table 2) displayed a mean difference of <4° in all the participants except Sub4. Additionally, the mean difference +2SD was <4° for all the subjects except Sub1 and Sub4. Subsequently, a comparison was conducted between the joint angle differences and range of motion. A characteristic abnormal posture of patients with PD often involves flexed knees and hips (Doherty et al., 2011). Consequently, the discrepancy in joint angle and range of motion was compared in the sagittal plane for the knee and lumbar joints, as representative examples. The maximum difference in joint angle was 5.2° for the knee joint and 5.0° for the lumbar joint, except for in Sub4 (Appendix in Supplementary material). The ranges of motion for the knee and lumbar joints were approximately 130° and 90°, respectively (Roach and Miles, 1991; Dvořák et al., 1995). In essence, the joint angle difference stood at merely 4.0% of the range of motion for the knee joint and 5.6% of that for the lumbar joint. Consequently, even in comparison to the range of motion, the joint angle difference between the simulation results and experimental outcomes using optimized parameters remained at < 1/10 of the joint motion range.

## 4. Discussion

We selected the muscle tone vectors identified in Section 3.3 as the ones responsible for actuating the joints of patients with PD, and proceeded to compare the resulting postures. As detailed in Section 3.4, the mean differences in the joint angles between the postures optimized to minimize sway and the postures of patients with PD were ≤ 1/10 of the range of joint motion. This outcome indicates that the extent of sway is smaller in the abnormal posture than in other postures.

Conversely, the mean + 2SD of the joint angle exceeded 4° only in Sub1 and Sub4. These variations are likely attributable to differences in disease severity among the patients. For instance, Sub1 exhibited the longest disease duration and a Hoehn & Yahr (H&Y) scale score of 3. Similarly, Sub4 exhibited the highest Unified Parkinson's Disease Rating Scale (UPDRS) Part III score compared with those of the other participants, as indicated in Table 1. These observations point to greater overall PD severity in Sub1 and more severe motor symptoms in Sub4 compared with that in the other participants. Furthermore, as illustrated in Figure 3, Subs 1 and 4 demonstrated more pronounced abnormal postures than the other participants. Reportedly, patients with advanced PD might experience alterations in soft tissue, muscles, and even the spinal cord, potentially leading to more pronounced postural deformities (Doherty et al., 2011). Thus, it is conceivable that, for Subs 1 and 4, maintaining a specific posture for an extended period could have contributed to skeletal deformation, thereby hindering their ability to assume different postures. The omission of skeletal deformation as a consideration in our study might have influenced the disparity between the experimental results and the simulation outcomes obtained with optimized parameters.

Herein, we calculated multiple muscle tone vectors based on the observed experimental postures. Additionally, the FB gains and postures of our computational model were adjusted using an optimization method for various muscle tone vectors. This adjustment allowed the musculoskeletal model to sustain the standing posture, and we sought to identify a posture with reduced sway starting from the experimental posture. By comparing the simulation results achieved using the optimized parameters to the experimental outcomes, we found that the muscle tone vectors with $\|u_{\text{ff}}|{|}^{2}$ values surpassing those of healthy participants yielded the closest resemblance to the experimental data. In essence, the posture and sway achieved through these larger $\|u_{\text{ff}}|{|}^{2}$ muscle tone vectors best replicate the characteristics of patients with PD. This observation aligns with prior research that highlights increased muscle tone among patients with PD compared with their healthy individuals (Burleigh et al., 1995). Furthermore, the disparities in joint angles between the optimized postures (which minimized sway) at these muscle tone vectors and the experimental postures were < 1/10 of the joint range of motion. These findings suggest that patients with PD who exhibit increased muscle tone experience reduced sway in their abnormal postures compared with other stances. Consequently, to effectively mitigate sway in the postural control of patients with PD, it might be crucial to maintain an abnormal posture with their increased muscle tone across the entire body. Therefore, the hypothesis of abnormal posture with an increased muscle tone leading to a smaller sway compared with that in other postures, including normal upright standing, under the sway minimization criterion may be effective for postural control in patients with PD.

Prior research has indicated variations in muscle tone among different patients, with some exhibiting higher muscle tone while others exhibiting lower muscle tone levels (Horak et al., 1992; Yamamoto et al., 2011). The findings of our study revealed that in all the participants, the muscle tone observed was generally higher than that in healthy individuals. Therefore, the results of this study may represent a strategy for patients with higher muscle tone. As mentioned in the Introduction section, prior experiments involving lidocaine administration to the external oblique muscle reported improvements in abnormal postures (Furusawa et al., 2015). In the same experiment, as the tilt of the tilt table approached 90°, abnormal posture became more pronounced alongside an increase in muscle tone (Furusawa et al., 2015). In addition, a model-based categorization of patients with PD revealed that the strategies they employ differ based on ankle flexibility, which is closely associated with muscle tone levels (Suzuki et al., 2020). Therefore, patients with lower muscle tone may not exhibit abnormal postures as prominently.

### 4.1. Limitation

Herein, we computed muscle tone vectors based on measured abnormal postures and used an optimization method to determine postures that minimized sway compared with other postures. As a result, the postures to minimize sway corresponded to abnormal postures. Therefore, the muscle tone vectors calculated for patients with PD in this study reflect abnormal postures. The precise reasons for the differences in muscle tone vectors between patients with PD and healthy individuals were not addressed within this study. Additional research into alterations in muscle tone vectors is warranted.

As previously mentioned, musculoskeletal anomalies have been associated with abnormal posture, particularly in patients with advanced or late-stage PD (Doherty et al., 2011). Herein, we replicated the postures of patients with PD using a musculoskeletal model. However, considering musculoskeletal deformities in patients who exhibited such postures was not feasible within our study. Gathering comprehensive skeletal system data for each participant to accurately simulate such deformities poses significant challenges. Nevertheless, a more comprehensive exploration of abnormal posture, especially in patients with later-stage of PD, could be achieved by focusing on established musculoskeletal deformities and conducting simulations based on these assumptions.

We focused on the static standing posture and did not delve into dynamic posture control, such as walking or walking initiation. Previous research has suggested that abnormal posture functions as a compensatory mechanism to enhance CoM movement at the beginning of walking (Jacobs et al., 2005). Investigating the influence of these postures on dynamic postural control would be valuable, and simulations involving actions such as walking initiation could provide further insights into abnormal postures.

We formulated the evaluation function in this study based on the assumption that minimizing the CoM velocity is essential during human standing posture control. However, other evaluation functions such as energy and muscle activity minimization are also considered in human posture control, but we did not use them in this study. Nevertheless, considering fall prevention, the reduction of CoM velocity is crucial, thereby making the evaluation function we used suitable for exploring our hypothesis.

Considering that abnormal postures occur because of increased muscle tone, it may be possible that the higher muscle tone leads to more abnormal postures. However, abnormal postures can manifest in diverse ways, encompassing factors such as foot width and body leaning. Consequently, defining the extent of postural abnormalities either qualitatively or quantitatively is challenging.

### 4.2. Conclusions and future perspectives

Herein, we examined the following hypothesis on abnormal posture during static standing: abnormal posture with increased muscle tone leads to smaller sway compared with that in other postures, including normal upright standing, under the sway minimization criterion. We used a computational model comprising musculoskeletal and neural controller models to test the proposed hypothesis. The norm of the muscle tone vector that could reproduce the most appropriate experimental posture and sway for patients with PD was larger than that for healthy individuals, consistent with the increased muscle tone in patients with PD. The average difference in the joint angles between the experimental posture and the posture to minimize sway was <4° except for one participant. The results of the computational model suggested that patients with PD with increased muscle tone display less sway in abnormal postures than in other postures. These findings suggest that once the muscle tone of patients with PD becomes that at an anterior leaning posture, they need to maintain an abnormal posture because their sway is greater in other postures compared with that in the abnormal posture. Given the importance of sway reduction in human postural control, it may be desirable to maintain abnormal postures more than the other postures and it may be necessary to alter muscle tone to change the standing posture. Therefore, the hypothesis described in this study is considered valid. In future studies, we intend to use the computational model used in this study to explore the interplay between walking initiation, disturbances, and abnormal posture. Additionally, we aim to delve into the connection between dopamine, the underlying cause of PD, and the parameters of the computational model.

## Data availability statement

The original contributions presented in the study are included in the article/Supplementary material, further inquiries can be directed to the corresponding author.

## Ethics statement

The studies involving humans were approved by the Ethics Committee at the National Center of Neurology and Psychiatry. The studies were conducted in accordance with the local legislation and institutional requirements. The participants provided their written informed consent to participate in this study.

## Author contributions

YO performed the simulations, analyzed the data, and drafted the manuscript. HT, MA, YT, and THan performed the experiments and acquired the data. HT, KK, THas, RC, AY, KT, THan, and JO reviewed the research methods. JO supervised the study. All authors discussed the results and reviewed the article. All authors contributed to the article and approved the submitted version.
